# FGFR3IIIS: a novel soluble FGFR3 spliced variant that modulates growth is frequently expressed in tumour cells

**DOI:** 10.1038/sj.bjc.6601249

**Published:** 2003-09-30

**Authors:** L-M Sturla, A E Merrick, S A Burchill

**Affiliations:** 1 Candlelighter's Children's Cancer Research Laboratory, Cancer Research UK Clinical Centre, St. James's University Hospital, Beckett Street, Leeds LS9 7TF, UK

**Keywords:** FGFR3IIIS, FGFR3IIIc, splice variant, tumours, soluble protein, dominant negative

## Abstract

Fibroblast growth factor receptor 3 (FGFR3) is one of four high-affinity tyrosine kinase receptors for the FGF family of ligands, frequently associated with growth arrest and induction of differentiation. The extracellular immunoglobulin (IgG)-like domains II and III are responsible for ligand binding; alternative usage of exons IIIb and IIIc of the Ig-like domain III determining the ligand-binding specificity of the receptor. By reverse transcriptase polymerase chain reaction (RT–PCR) a novel FGFR3IIIc variant FGFR3IIIS, expressed in a high proportion of tumours and tumour cell lines but rarely in normal tissues, has been identified. Unlike recently described nonsense transcripts of FGFR3, the coding region of FGFR3IIIS remains in-frame producing a novel protein. The protein product is coexpressed with FGFR3IIIc in the membrane and soluble cell fractions; expression in the soluble fraction is decreased after exposure to bFGF but not aFGF. Knockout of FGFR3IIIS using antisense has a growth-inhibitory effect *in vitro*, suggesting a dominant-negative function for FGFR3IIIS inhibiting FGFR3-induced growth arrest. In summary, alternative splicing of the FGFR3 Ig-domain III represents a mechanism for the generation of receptor diversity. FGFR3IIIS may regulate FGF and FGFR trafficking and function, possibly contributing to the development of a malignant phenotype.

The fibroblast growth factor (FGF) signal transduction occurs via a family of low- and high-affinity receptors. The high-affinity receptors are tyrosine kinase receptors encoded by four genes, with alternative splicing of their respective mRNAs generating many fibroblast growth factor receptor (FGFR) isoforms. They rely on the same core structure of an extracellular domain comprising an amino terminal hydrophobic signal peptide followed by three immunoglobulin (Ig)-like loops, a single hydrophobic transmembrane sequence and an intracellular split-tyrosine kinase domain. The ligand-binding site is confined to the extracellular Ig-like domains II and III (Avivi *et al*, 1993). The c-terminal half of Ig-domain III is encoded by two separate exons, exon 8 (IIIb) and exon 9 (IIIc); alternative usage of these exons determines the ligand-binding specificity of the receptor. The receptors and their respective alternatively spliced isoforms are expressed in a cell- and tissue-specific manner, important for initiating specific responses to the ubiquitously expressed FGF family of growth factors. It is now evident that the inappropriate expression of some isoforms can contribute to malignant progression (Morrison *et al*, 1994, Yamaguchi *et al*, 1994).

FGFR3 is normally expressed in the tissues of the central nervous system, brain, kidney and testis, but not in the spleen, heart or muscle (Cancilla *et al*, 1999; Plowright *et al*, 2000). Mis-sense mutations in the transmembrane region of FGFR3 result in constitutive activation and the autosomal dominant heritable disorders of skeletal development such as thanatophoric dwarfism, achondroplasia and Crouzon syndrome (Tavormina *et al*, 1995; Naski *et al*, 1996; Bonaventure *et al*, 2001). The phenotype observed in dwarfism suggests that the dysregulation of FGFR3 could impair cell growth and differentiation (Su *et al*, 1997; Legeai-Mallet *et al*, 1998; Sahni *et al*, 1999), knockout mouse studies confirming an inhibitory role for FGFR3 in bone growth (Colvin *et al*, 1996; Deng *et al*, 1996). However, FGFR3 may regulate differentiation (Kobrin *et al*, 1993; Kanai *et al*, 1997) or apoptosis (Plowright *et al*, 2000) dependent on the tissue type, consistent with a multifunctional role of FGFR3. FGFR3 normally exists in two forms, FGFR3IIIc and FGFR3IIIb, which arise following alternative splicing in which either exon 8 or 9 respectively are skipped. Little is known about FGFR3IIIc and its isoforms, although it is expressed in ovarian cancers where it is activated by FGF8 (Valve *et al*, 2000). However, FGFR3IIIb is the predominant form expressed in epithelial cells and epithelial-derived tumours such as colorectal cancer (Murgue *et al*, 1994; Jang *et al*, 2000). Recent studies suggest that the dysregulation of FGFR3IIIb mRNA splicing generates aberrant transcripts that may confer a selectable survival or growth advantage for colorectal cancer cells (Jang *et al*, 2000). Typically, the predicted transcription products exhibit a frame-shift and premature termination in exon 10, so no functional protein product is translated (Jang *et al*, 2000). Where mutations of FGFR3 are frequent events, for example, in bladder cancer (Cappellen *et al*, 1999; Billerey *et al*, 2001; van Rhijn *et al*, 2001; Sibley *et al*, 2001), nonsense mutations inserted within its coding region may result in altered splice site selection (Valentine, 1998). This is unlikely to be the case for the majority of cancers where mutations of FGFR3 are rare (Intini *et al*, 2001; Karoui *et al*, 2001; Sibley *et al*, 2001), although FGFR3 may be inactivated by aberrant-splicing and activation of cryptic splice donor sites (Jang *et al*, 2000). In this study we have identified and characterised a novel FGFR3IIIc variant, FGFR3IIIS, which is frequently expressed in tumorigenic but rarely in nontumorigenic cells. This variant is identical to FGFR3IIIc except for a 336 bp deletion resulting in loss of exons 9 and 10, and a 30 bp deletion in exon 7. FGFR3IIIS mRNA encodes a protein product that is co-expressed with full-length FGFR3IIIc in the membrane and soluble cell fraction. The expression and localisation of FGFR3IIIS and FGFR3IIIc are modulated by exposure to bFGF but not aFGF. FGFR3IIIS is not phosphorylated following exposure to bFGF or aFGF, but it may modulate dose-dependent ligand-induced phosphorylation of FGFR3IIIc. Furthermore, knockout of FGFR3IIIS decreases viable cell number, consistent with a dominant-negative role for FGFR3IIIS inhibiting FGFR3IIIc-induced growth arrest and differentiation.

## MATERIALS AND METHODS

### Cell lines

The following 19 cell lines (all cell lines were of human origin except the PC12 cells) were studied—two neuroblastoma (SK-N-SH and IMR-32), six tumours of the Ewing's sarcoma family (ESFTs)(TC-32, RD-ES, A673, SK-N-MC, SKES1 and TTC-466), a neurofibrosarcoma (ST118), two rhabdomyosarcoma (RH30 and CCL136), two bladder cancer (RT112 and EJ), a melanoma (VUP), prostate carcinoma (LNCAP), a colorectal carcinoma (HT29), a breast cancer (MCF7), a myeloma (U266) and a rat adrenal pheochromocytoma (PC12). TC-32 and RD-ES cells were a kind gift from Dr J Toretsky (National Institute of Cancer, Bethesda, USA), and TTC-466 was a gift from Dr PHB Sorenson (British Columbia Children's Hospital, Vancouver, Canada). All other cell lines were obtained from the American Tissue Culture Collection. Cell lines were grown in standard growth media plus 10% FCS at 37°C in 5% CO_2_: 95% air; growth media for TTC-466 and U266 cells was supplemented with 10% conditioned media.

### Tumour samples

A total of 30 tumours were studied; nine neuroblastoma, 12 ESFTs, two Wilms' tumours, two rhabdomyosarcomas, one astrocytoma and four medulloblastomas. All patients were treated at St James's University Hospital between 1992 and 1998. Histological and genetic diagnosies were made according to conventional criteria. All tumour specimens were obtained in accordance with the Leeds Teaching Hospital Trust Ethics Committee; samples were taken at diagnosis, with the exception of one neuroblastoma taken post-treatment, one neuroblastoma and one Ewing's sarcoma taken at relapse. Tumour biopsies were snap-frozen in liquid nitrogen at the time of surgery and stored at −80°C prior to analysis.

### Detection of FGFR3 splice-variants by reverse transcriptase–polymerase chain reaction (RT–PCR)

The total cellular RNA from cultured cells, tumours and tissues was isolated using Ultraspec™ (Biogenesis, Poole, UK), and amplified by reverse transcriptase-polymerase chain reaction RT–PCR (Burchill *et al*, 1994) for FGFR3. Primer sequences for PCR were chosen within exons 3 and 4 to amplify the first Ig-like loop of the extracellular domain (5′- ATTCGGGATGTGGAGCTGGAAGTGC-3′, 5′- TGAAACATTGACGGAGAAGTAGGTG-3′; primer set 1) and within exons 6 and 12 (5′-GAGAACAAGTTTGGCAGCATCC-3′, 5′-GCCCGAGACAGCTCCCATTTGG-3′; primer set 2) to amplify the third Ig-like loop and the transmembrane/tyrosine kinase domain.

RNA isolated from MCF-7 cells was used as a positive control; a negative control containing no reverse transcriptase was included to confirm that the amplified products were generated from cDNA rather than contaminating genomic DNA (reverse transcriptase negative control) and a PCR negative control in which there was no nucleic acid was included to control for contamination (water control). Further analysis of the FGFR3IIIc variant in normal cDNAs and RNAs was made by amplification using a primer specific to FGFR3IIIS sequence (exon 7/11 splice site), paired with a primer at the 5′ (exon 2; 5′-GACGCCGCGGCCCCCGCCCCCGCCA -3′) or 3′ (exon 17; 5′-AGAGCAGGACCCCAAAGGACCAGAC -3′; primer set 3) ends of the published wild-type sequence. Amplified products (30 *μ*l) were separated by agarose gel electrophoresis (1% agarose in 1 × TBE), gels were stained with ethidium bromide (0.5 *μ*g ml^−1^) and visualised under UV light. Products were excised from the gel and DNA extracted using the QIAquick gel extraction kit (QIAGEN Ltd, Crawley, UK). Each amplified product (approximately 50 ng) was sequenced using the ABI PRISM Big dye terminator kit (Perkin-Elmer, Applied Biosciences Ltd, Warrington, UK) and an ABI 377 automated sequencer. PCR primers (1.6 pmol per reaction) were used as forward and reverse sequencing primers.

### Southern blotting of PCR products

PCR products, separated by size on agarose gels, were transferred on to a Hybond-N^+^ nitrocellulose membrane (Amersham, Biosciences (UK) Ltd, Bucks, UK) and probed with an FGFR3IIIS specific riboprobe. The riboprobe was generated by cloning the FGFR3IIIS PCR product amplified from MCF7 cells into the pCR®2.1-TOPO vector (Invitrogen, Leek, Netherlands) and transcribing using the Riboprobe® *in vitro* transcription system (Promega, Southampton, UK). Membranes were prehybridised at 42°C for 2 h in a hybridisation solution (50% formamide, 0.02% SDS, 0.1% *N*-laurylsarcosine, 5 × SSC, 5% blocking reagent, 50 *μ*g ml^−1^ denatured salmon testis DNA). Hybridisation was carried out with 1 × 10^6^ c.p.m. FGFR3IIIS riboprobe/ml of the hybridisation buffer overnight at 42°C. After hybridisation, membranes were sequentially washed at room temperature (RT) twice for 30 min in 2 × SSC, 0.1% SDS, followed by two washes in 1 × SSC, 0.1% SDS.

### Detection of FGFR3IIIS in normal human tissues

cDNA reverse transcribed from 24 different human tissues (brain, heart, kidney, spleen, liver, colon, lung, small intestine, muscle, stomach, testis, placenta, salivary gland, thyroid gland, adrenal gland, pancreas, ovary, uterus, prostate, skin, peripheral blood lymphocytes, bone marrow, foetal brain and foetal liver) in a 96-well plate format (Origene Technologies, Rockville, MA, USA) was amplified by PCR using the conditions described above and primer set 3. PCR products (20 *μ*l) were separated by electrophoresis on a 1% agarose gel in 1 × TBE. Gels were stained with ethidium bromide (0.5 *μ*g ml^−1^) and visualised under UV light. The quality of cDNA for PCR was confirmed by amplification for the house-keeping gene *β*_2_-microglobulin.

### Protein expression and localisation of FGFR3IIIc and FGFR3IIIS

Cells were lysed in lysis buffer A (50 mM NaCl, 0.05%. (v v^−1^) NP-40, 0.5% (w v^−1^) sodium deoxycholate, 100 *μ*M phenylmethyl-sulponyl fluoride (Sigma, Poole, UK)) and incubated on ice for 20 min with occasional vortexing. Cell debris was pelleted by centrifugation at 11 600 g for 10 min at 4°C and the supernatant lysate was retained. The protein content of lysates was estimated using the Bio-rad *DC* Protein Assay (Bio-rad, Hemel Hempstead, UK). Protein samples (20–60 *μ*g) were resolved by SDS–PAGE in an SDS electrophoresis buffer (25 mM Tris-HCl, pH 8.3, 250 mM glycine, 0.1% (w v^−1^) SDS) at 200 V, and electrophoretically transferred on to a Hybond C membrane (Amersham). To confirm equal loading of protein, duplicate samples were separated by SDS–PAGE and stained with silver salts (Pharmacia, St Albans, UK).

All blocking, antibody incubation and wash steps were carried out with gentle agitation. The non-specific binding of antibodies was reduced by incubating nitrocellulose membranes in 5% (w v^−1^) nonfat dried skimmed milk (Marvel; NHS supplies authority) in Tween 20-Tris-buffered saline (TTBS; 20 mM Tris-HCl pH 7.5, 500 mM NaCl, 0.05% (v v^−1^) Tween 20 (Sigma)) for 2 h at RT. Membranes were incubated with a polyclonal antibody raised against a peptide to the carboxyl terminus of FGFR3 (SC-123, 1 : 200; Santa Cruz Biotechnology Inc., Santa Cruz, California, USA) in TTBS containing 1% (w v^−1^) nonfat dried milk for 2 h at RT; the signal was detected using an HRP-conjugated secondary antibody (1 : 2000; Dako, High Wycombe, UK) for 45 min at RT and enhanced chemiluminescence (ECL, Amersham Biosciences (UK) Ltd, Bucks, UK). A negative control blot was incubated with the secondary antibody alone, to check for nonspecific binding of the secondary antibody. The high degree of homology between FGFR3IIIc and FGFR3IIIS has so far prevented the production of antibodies specific to these individual proteins; however, the specificity of SC123 binding to FGFR3-related proteins was evaluated by preincubation of the antibody with blocking peptides to FGFR1 (SC-121P 50 *μ*g ml^−1^, Santa Cruz Biotechnology Inc.) or FGFR3 (SC-123P 50 *μ*g ml^−1^, Santa Cruz Biotechnology Inc.) for 10 min at RT prior to incubation with the Western blot. Total protein was determined using a pan-ERK mouse monoclonal antibody (0.25 *μ*g ml^−1^; BDBioscience-Transduction Laboratories, Cowley, UK).

The effect of bFGF (10 μml^−1^) and aFGF (10 ng ml^−1^) in the presence of heparin (10 U ml^−1^), on the expression of FGFR3IIIc and FGFR3IIIS after 48 h was examined in seven cell lines (SK-N-SH, RD-ES, SKES1, TC-32, TTC466, A673 and MCF7) by Western blot. The localisation of both proteins was investigated in TC-32 cells under normal growth conditions and in the presence of bFGF (10 ng ml^−1^) by differential centrifugation and Western blot. Cells were lysed in lysis buffer B (10 mM Tris-HCl pH7.4, 0.001 M EDTA, 10 g ml^−1^ aprotinin, 100 g ml^−1^ leupeptin), an aliquot was taken for total protein analysis and the nuclear, mitochondrial, plasma membrane, ribosomal and soluble protein fractions were prepared (Figure 4D). The identity of the mitochondrial fraction was confirmed by blotting for cytochrome *c* (0.4 *μ*g ml^−1^, 17 kDa; PharMingen, San Diego, CA, USA) and that of the nuclear and ribosomal fractions for Nucleoporin p62 ( 1: 1000, 62 kDa; Transduction Laboratories).

### Activation of FGFR3 and FGFR1 following exposure to aFGF and bFGF

The effect of aFGF (0.1–10 ng ml^−1^) and bFGF (0.1–10 ng ml^−1^) on tyrosine phosphorylation of FGFR1 and FGFR3 in TC-32 cells was examined by immunoprecipitation and Western blot. TC-32 cells were grown in low serum conditions (0.5% FCS in RPMI 1640 media) for 24 h, and then treated with either aFGF (0.1, 1 or 10 ng ml^−1^) or bFGF (0.1, 1 or 10 ng ml^−1^) in low serum media containing heparin (10 U ml^−1^) for up to 30 min at 37°C. Heparin was included in conditions for FGF treatment as its molecular association with FGF and its receptors is reported to be essential for biological activity (Rapraeger *et al*, 1991; Lin *et al*, 1999). Flasks were placed in ice, cells were washed in cold PBS and lysates were prepared in lysis buffer (50 mM HEPES (pH 7.5), 100 mM NaCl, 1 mM EGTA, 1 mM DTT, 10 mM MgCl_2_, 1% (v v^−1^) Triton X-100, 10 *μ*g ml^−1^ aprotinin, 10 *μ*g ml^−1^ leupeptin, 1 mM sodium orthovanadate (Sigma), 25 mM sodium fluoride (Sigma)). The cell lysate (200 *μ*g) containing 10% (w v^−1^) SDS was denatured by heating at 100°C and immediately placed on ice. Rabbit polyclonal antibodies to FGFR1 (5 *μ*g SC121; Santa Cruz Biotechnology Inc) or FGFR3 (5 *μ*g SC123; Santa Cruz Biotechnology Inc.) and 30 *μ*l of protein A sepharose CL-4B (50% (v v^−1^) slurry in 20 mM sodium phosphate, pH 7.0; Pharmacia) were added to the cooled cell lysate and incubated overnight with rotation at 4°C. At 4°C sepharose was pelleted by centrifugation at 11 600 g for 5 min, the supernatant was discarded and the pellet was washed three times in 1 ml of ice-cold PBS containing 0.1% (w v^−1^) SDS. Immunoprecipitated proteins were isolated by heating samples for 5 min at 100°C in SDS sample buffer (100 mM Tris-HCl pH 6.8, 200 mM DTT, 4% (w v^−1^) SDS, 0.2% (w v^−1^) bromophenol blue, 20% (v v^−1^) glycerol) and analysed by SDS–PAGE and Western blot using anti-phosphotyrosine monoclonal IgG (0.5 *μ*g ml^−1^; Upstate Biotechnology (UK) Ltd, Bucks, UK) as above, except that membranes were blocked in 1% (w v^−1^) BSA in TTBS and both primary and secondary antibodies were diluted in 0.1% (w v^−1^) BSA in TTBS. The cell extract from A431 after treatment with the epidermal growth factor was included as a positive control for the tyrosine phosphorylation antibody. The optimal concentration of all antibodies was determined empirically. An immunoprecipitation control with no primary antibody was used to determine nonspecific binding of proteins to the sepharose.

The activation of ERK was not measured directly, but by immunoblotting for total ERK protein and looking for a shift in mobility as the protein is activated. The total cell lysate (50 *μ*g) was separated on a 15% (w v^−1^) low bis-SDS-polyacrylamide gel; the final percentage of acrylamide in the resolving gel had a ratio of acrylamide:*N*,′*N*-methylene bisacrylamide of 200 : 1 (Anachem). The anti-pan-ERK antibody was used at 0.25 *μ*g ml^−1^ (BD Biosciences-Transduction Laboratories Cambey, UK).

### Knockout of FGF3IIIS in TC-32 cells

The expression of the FGFR3IIIS protein was reduced in TC-32 cells using one HPLC-purified antisense 24-mer oligonucleotide targeted to the breakpoint between exons 7 and 11 of FGFR3IIIS (exon 7 ACCGTGCTCAAG:GTGTCCCTGGAG exon 11; : = breakpoint) to maximise the inhibition of FGFR3IIIS translation compared to that of FGFR3IIIc. Conditions for optimum cellular uptake of antisense oligonucleotides were established using an FITC-labelled oligonucleotide (Biognostik, TCS Biologicals, Buckingham, UK; Schlingensiepen *et al*, 1997). In brief, TC-32 cells (2.5 × 10^5^) were plated in primeria six-well plates and allowed to adhere overnight in normal growth media plus 10% FCS. At time zero (0 h), media was replaced with normal media plus 1% FCS containing (i) HPLC-purified antisense FGFR3IIIS 24-mer at 1 *μ*M, (ii) HPLC-purified random scrambled oligonucleotide 24-mer at 1 *μ*M or (iii) no oligonucleotide. Oligonucleotides were replenished in cell cultures 24 and 48 h after initial incubation (0 h). Cells were harvested 24, 48 and 72 h after incubation and an aliquot from each well was taken for viable cell counting using a Neubauer haemocytometer and the trypan blue-exclusion assay. The effect of antisense FGFR3IIIS and random scrambled oligonucleotides on viable cell number with time was evaluated using ANOVA and *posthoc* tests (Tukey–Kramer multiple comparisons test). The remaining cells for each condition were pooled and cell lysates were prepared for SDS–PAGE and Western blot using FGFR3 antibodies (see above) to monitor the effect of the oligonucleotides on FGFR3IIIS expression.

## RESULTS

### Detection of FGFR3 by RT–PCR

**Figure 1:**
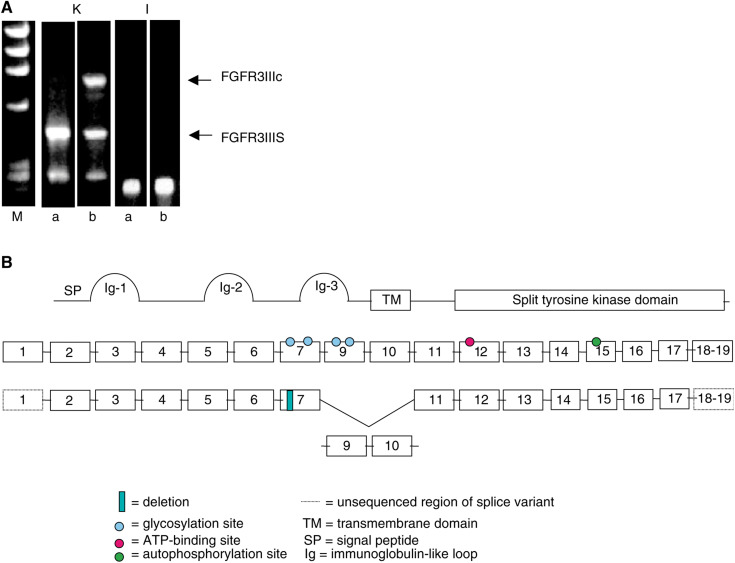
Identification and characterisation of a novel FGFR3IIIc variant, FGFR3IIIS. (**A**) The FGFR3 extracellular domain, first Ig-like loop (I) and the transmembrane/intracellular domain (K) was amplified in the breast carcinoma MCF-7 (a) and Ewing's sarcoma TC-32 (b) cell lines by RT–PCR. *M*=φX174 RF DNA/*Hae*III molecular weight marker. (**B**) Diagrammatical representation of FGFR3IIIc, FGFR3IIIS and the domains that each exon codes for. The split tyrosine-kinase domain is represented as one domain, since it is not known as to which exon codes for the region between the two domains. Sequence analysis of FGFR3IIIS demonstrated loss of exons 9 (encoding the second half of the third Ig-like domain) and 10 (coding for the transmembrane domain) and a 30 bp deletion in exon 7 (within the region between the second and third Ig-like loops, including a potential glycosylation site). Primers were designed in exons 3 and 4 to amplify the first Ig-like loop of the extracellular domain (primer set 1) and within exons 6 and 12 (primer set 2) to amplify the third Ig-like loop and the transmembrane/tyrosine kinase domain.

A single PCR product was generated in the MCF-7 breast carcinoma (a) and TC-32 ESFT (b) cell lines (Figure 1A

; I) using primer set 1 designed to amplify the first Ig-like loop of the extracellular domain (Avivi *et al*, 1993). A product of the same size and identity was also detected in the neuroblastoma cell lines SK-N-SH and IMR-32, and the ESFT cell lines RD-ES, A673 and SK-N-MC studied (results not shown).

Primer set 2 designed to amplify across the third Ig-like loop of the extracellular domain, the transmembrane domain and part of the tyrosine kinase domain generated up to three distinct PCR products (Figure 1A; K). The expected full-length product (FGFR3IIIc) was detected in the TC-32 ESFT cells, but not in the MCF-7 cells. Two smaller products were detected in the MCF-7 and TC-32 cell line RNA; the most abundant of these (FGFR3IIIS) was isolated for further characterisation.

### Characterisation of an alternatively spliced FGFR3IIIc gene product, FGFR3IIIS

The sequence of the FGFR3IIIS PCR product was 100% identical to the full-length FGFR3IIIc except for a 336 bp deletion resulting in the loss of exons 9 and 10 and a 30 bp deletion in exon 7 (Figure 1B). These deletions resulted in an in-frame coding region, consistent with the hypothesis that this is a splice-variant of FGFR3IIIc. The sequence identity of FGFR3IIIS was confirmed in MCF7 breast carcinoma and the Ewing's sarcoma cell lines studied. Exon 9 codes for the second half of the third Ig-like loop, suggesting loss of the loop structure in this region, and exon 10 encodes the transmembrane region. The 30 bp deletion in exon 7 results in the loss of a region between the second and third Ig-like loops, and includes a potential glycosylation site (Figure 1B).

### Expression of FGFR3IIIS mRNA in normal human tissue, cultured tumour cell lines and tumour tissue

**Figure 2:**
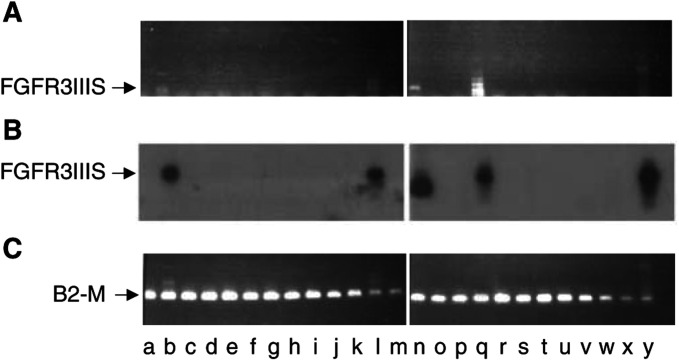
FGFR3IIIS is rarely expressed in normal human tissue. Expression of the FGFR3IIIS was examined by RT–PCR using primer set 3 (**A**) and Southern blotting using an FGFR3IIIS-specific probe (**B**) in a number of normal human tissues. FGFR3IIIS was amplified using a primer specific to the FGFR3IIIS sequence at the exon seven out of 11 splice site, paired with a primer at the 5′ (exon 2) or 3′ (exon 17) ends of the published wild-type FGFR3IIIc sequence. Low-level expression of the FGFR3IIIS was detected in the heart (b), placenta (l) and ovary (q). The probe also bound nonspecifically (product identified in the reverse transcriptase negative control; not shown) to a smaller product in the thyroid gland (n). RT–PCR for the house-keeping gene *β*_2_-microglobulin (*β*_2_-m) confirmed the quality of cDNA for amplification (**C**) a, brain; b, heart; c, kidney; d, spleen; e, liver; f, colon; g, lung; h, small intestine; i, Muscle; j, stomach; k, testis; l, placenta; m, salivary gland; n, thyroid gland; o, adrenal gland; p, pancreas; q, ovary; r, uterus; s, prostate; t, skin; u, peripheral blood lymphocytes; v, bone marrow; w, foetal brain; x, foetal liver: RNA from the MCF-7 cell line was included as a positive control (y).

FGFR3IIIS was detected by PCR (using primer set 3) in heart (b), placenta (l) and ovary (q) cDNAs at low levels (Figure 2A

); this was confirmed by Southern blot using an FGFR3IIIS-specific riboprobe (Figure 2B). RT–PCR on newly prepared total RNA extracted from the human adrenal, brain, kidney, liver, placenta and pericardium confirmed the expression of FGFR3IIIS only in the placenta and pericardium (results not shown). The quality of RNA and cDNA for RT–PCR and PCR, respectively, was confirmed by amplification for the house-keeping gene *β*_2_-microglobulin (Figure 2C).

**Figure 3:**
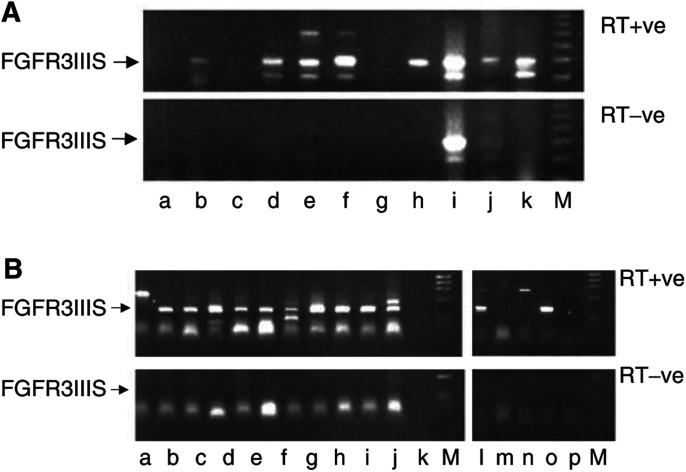
Expression of FGFR3IIIc and FGFR3IIIS in tumour cell lines and primary tumours. Expression of FGFR3IIIc (detected by RT–PCR using primer set 2) was rare in the tumour cell lines and tumours examined; however, the splice variant FGFR3IIIS was expressed in 57% (17 out of 30) of tumours (a) and 79% (15 out of 19) of the tumour cell lines (b) examined. The quality of RNA from cell lines and tumours was confirmed by amplification for the house-keeping gene *β*_2_-microglobulin (results not shown). (**A**) RT-PCR for FGFR3IIIS (a) rhabdomyosarcoma, (b) Wilms' tumour, (c) astrocytoma, (d and j). neuroblastoma, (e–h) Ewing's sarcoma, (i) medulloblastoma, (k) MCF7 cell line. (**B**) FGFR3IIIS RT-PCR for (a) U266, (b) IMR-32, (c) RD-ES, (d) TC-32, (e) SK-N-MC, (f) TTC-466, (g) ST118, (h) RT112, (i) LNCAP, (j) CCL136, (k) VUP, (l) RH30, (m) PC12, (n) EJ, (o) HT29, (p) MCF7 cell lines. M=molecular weight markers; RT+ve=reverse transcriptase enzyme present; RT-ve=no reverse transcriptase enzyme included in the reaction; negative control.

FGFR3IIIS was detected by RT–PCR (using primer set 2) in a high proportion of human tumour cell lines and primary tumours. Interestingly, it was expressed in a higher proportion of primary tumours (57%; 17 out of 30) than the full-length FGFR3IIIc (13%; four out of 30). Full-length FGFR3IIIc was detected in three out of the 12 Ewing's sarcomas and one of the two Wilms' tumours; it was not detected in the neuroblastoma, rhabdomyosarcoma, astrocytoma or medulloblastoma samples. However, FGFR3IIIS was detected in nine of the 12 Ewing's sarcomas, two of the nine neuroblastomas, three of the four medulloblastomas, both of the Wilms' tumours and one of the rhabdomyosarcomas (Figure 3

). One Ewing's sarcoma and one neuroblastoma taken at relapse expressed FGFR3IIIS but not FGFR3IIIc; FGFR3IIIS and FGFR3IIIc were not detected in a neuroblastoma taken post-treatment (although the RNA was successfully amplified for *β*_2_-microglobulin).

FGFR3IIIS was also detected in 15 out of 19 cell lines; the IMR-32 and SK-N-SH (neuroblastoma), RD-ES, TC-32, SK-N-MC, SKES1 and TTC-466 (tumours of the Ewing's sarcoma family), ST118 (neurofibrosarcoma), RT112 (bladder carcinoma), LNCAP (prostate carcinoma), CCL136 and RH30 (rhabdomyosarcomas), VUP (melanoma), HT29 (colorectal carcinoma) and MCF7 (breast carcinoma) cell lines were all positive (Figure 3B). FGFR3IIIS was not detected in PC12, EJ, A673 and U266 cell lines.

### FGFR3IIIS mRNA encodes a protein that is coexpressed with FGFR3IIIc

FGFR3IIIS mRNA has 100% sequence homology with that of FGFR3IIIc, except in this novel variant 30 bp of exon 7, and the complete exons 9 and 10 sequence are deleted. The missing sequence causes exon 7 to be directly spliced to exon 11, but leaves the coding region in-frame, suggesting that FGFR3IIIS may code for a novel FGFR3 protein. This is in direct contrast to the aberrant FGFR3 cDNAs, FGFR3 AT-II and FGFR3 AT-I identified in colorectal cancer cells, where the predicted translation products exhibit frame-shifts and a premature termination codon in exon 10 (Jang *et al*, 2000).

**Figure 4:**
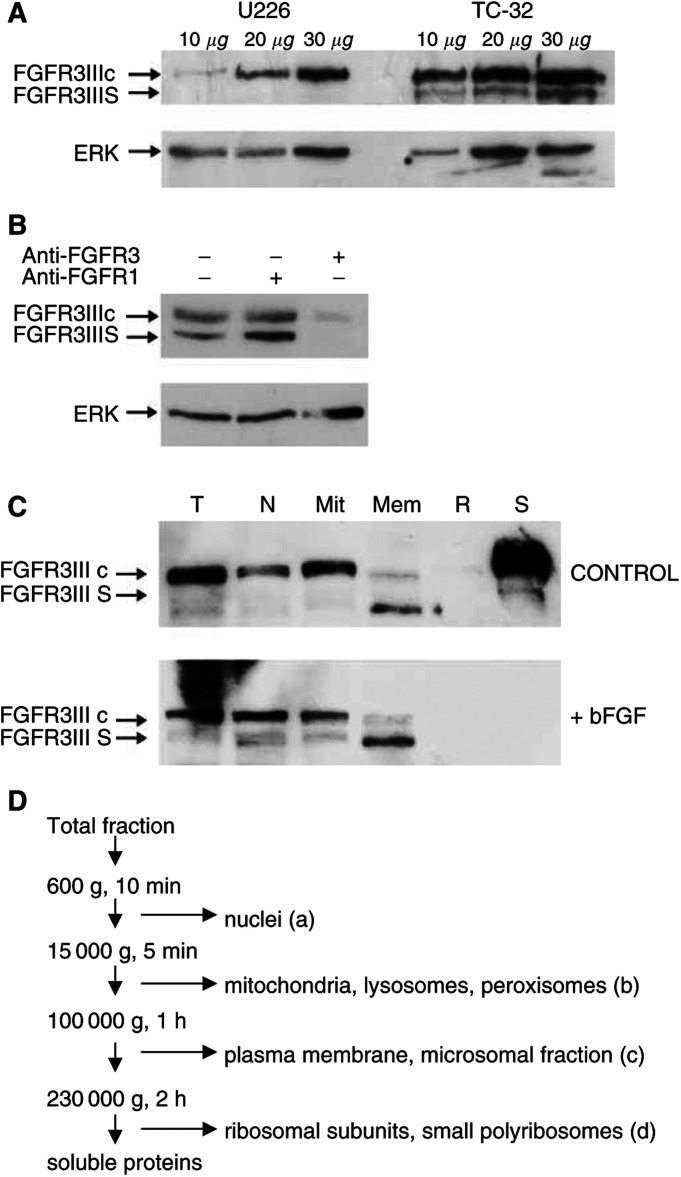
Variant FGFR3IIIS mRNA encodes a protein product that is differentially expressed in TC-32 and U266 tumour cell lines, and coexpressed with FGFR3IIIc in the membrane and soluble cell fractions. (**A**) TC-32 cells expressed both FGFR3IIIS and FGFR3IIIc; the expression was greater as the amount of cell extract analysed was increased. U226 cells did not express FGFR3IIIS, but did express FGFR3IIIc. Protein loading was confirmed by probing for ERK (42 kDa). (**B**) The identity of FGFR3IIIS and FGFR3IIIc was confirmed by incubating the TC-32 cell extract with a blocking peptide to FGFR1 (anti-FGFR1) or FGFR3 (anti-FGFR3) prior to SDS–PAGE and Western blot. In the presence of blocking peptide with FGFR3, the expression of FGFR3IIIS and FGFR3IIIc as detected on Western blot was decreased; incubation with blocking peptide for FGFR1 had no effect on expression of either protein. Protein loading was confirmed by probing for ERK (42 kDa). (**C**) FGFR3IIIS protein was coexpressed with full-length FGFR3IIIc in the soluble and membrane fractions. Following treatment with bFGF (10 ng ml^−1^) for 48 h, FGFR3IIIS and FGFR3IIIc proteins were not detected in the soluble fraction. The significance of this is not clear. T=total protein extract. Fractions: N=nuclear, Mit=mitochondrial, Mem=membrane, R=ribosomal, S=soluble. (**D**) Summary of the differential centrifugation method to prepare subcellular fractions.

Consistent with this hypothesis, a protein of approximately 110 kDa was identified on Western blot using the SC123 FGFR3 polyclonal antibody in the cell extract from TC-32 cells (Figure 4A

). This protein product was not detected in U266 cells (Figure 4A), correlating with the absence of the FGFR3IIIS splice-variant mRNA in these cells (Figure 3B). The FGFR3IIIS protein was expressed in 37% (seven out of 19) of the cell lines studied; very high levels were detected in RT112 cells, moderate expression in TC-32, SK-N-MC, HT-29 and MCF7 cells and low expression in RD-ES cells. Binding of SC123 to wild-type FGFR3IIIc and this 110 kDa protein was not inhibited by incubation with an FGFR1-blocking peptide, but was decreased following incubation with an FGFR3-blocking peptide (Figure 4B), suggesting SC123 was binding specifically to FGFR3 proteins. The FGFR3IIIS protein product (110 kDa) was predominantly expressed in the membrane fraction of TC-32 cells (Figure 4C), suggesting that it may regulate biological activity and signal transduction of FGFR3 and its variants in these malignant cells.

### Decreased soluble FGFR3IIIc and FGFR3IIIS protein expression after exposure to bFGF but not aFGF

Although FGFR3IIIS is predominantly expressed in the membrane fraction, it is also co-expressed with FGFR3IIIc in the soluble fraction of TC-32 cells under normal growth conditions (Figure 4C, CONTROL). Following treatment with bFGF (10 ng ml^−1^) for 48 h, the expression of soluble FGFR3IIIc and FGFR3IIIS proteins was lost when detected by Western blot (Figure 4C, +bFGF), although the expression of the membrane-associated FGFR3IIIS protein was unchanged. Following exposure of TC-32 cells to lower concentrations of bFGF (0.1–1 ng ml^−1^) there was no change in the expression or localisation of FGFR3IIIS or FGFR3IIIc (results not shown). Cellular levels and subcellular localisation of FGFR3IIIS and FGFR3IIIc were unchanged in TC-32 cells after treatment with aFGF (0.1–20 ng ml^−1^)(results not shown).

### FGFR3IIIc but not FGFR3IIIS is phosphorylated after treatment with bFGF or aFGF

**Figure 5:**
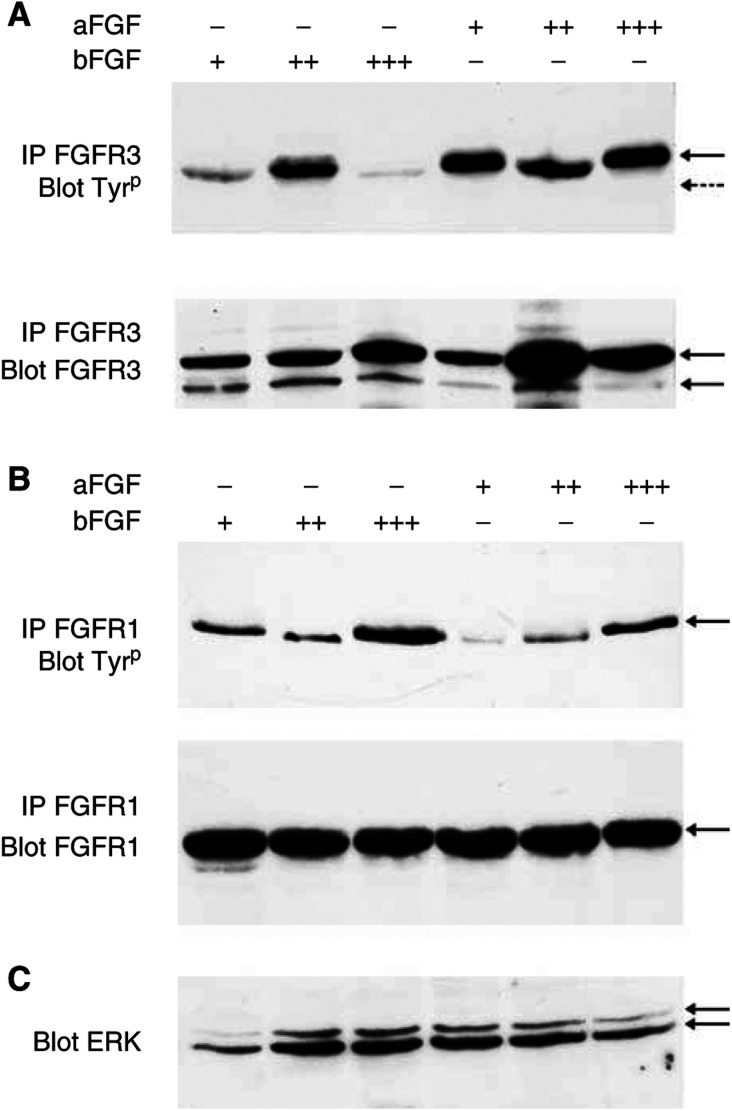
Phosphorylation of FGFR3IIIc but not FGFR3IIIS after exposure of TC-32 cells to bFGF or aFGF. (**A**) After treatment of TC-32 cells with bFGF (0.1 ng ml^−1^; +) or bFGF (1 ng ml^−1^ ;++) for 10 min FGFR3IIIc protein, immunoprecipitated (IP) with FGFR3 antibodies, was tyrosine phosphorylated (← ;IP FGFR3 Blot Tyr^p^). At higher doses of bFGF (10 ng ml^−1^; +++), FGFR3IIIc was poorly phosphorylated. Exposure to aFGF (0.1–10 ng ml^−1^) induced tyrosine phosphorylation of FGFR3IIIc. There was no evidence of tyrosine phosphorylation of FGFR3IIIS (← - -). Immunoprecipitation and Western blotting for FGFR3 proteins (IP FGFR3 Blot FGFR3) confirmed the presence of FGFR3IIIc and FGFR3IIIS. (**B**) FGFR1 (←) was phosphorylated after exposure to bFGF (0.1, 1 or 10 ng ml^−1^; +, + + or +++) or aFGF (0.1, 1 or 10 ng ml^−1^; +, + + or +++) for 10 min demonstrated by immunoprecipitation for FGFR1 and Western blotting for tyrosine-phosphorylated proteins (IP FGFR1 Blot Tyr^p^). Immunoprecipitation and Western blotting confirmed the presence of the total the FGFR1 protein (IP FGFR1 Blot FGFR1). (**C**) Following exposure of TC-32 cells to bFGF or aFGF the intracellular signalling protein ERK was activated, demonstrated by a mobility shift of the protein detected by Western blot (Blot ERK).

Phosphorylation of the FGFR3-precipitated protein occurred in TC-32 cells after treatment with bFGF (0.1–1 ng ml^−1^; +, ++) for 5–15 min (Figure 5A

). The size of the precipitated, phosphorylated band was consistent with the identity of FGFR3IIIc (Figure 5A ←); FGFR3IIIS appeared not to be phosphorylated following exposure to bFGF or aFGF (Figure 5A ← - -). At higher doses of bFGF (10 ng ml^−1^, +++), phosphorylation of FGFR3 precipitated proteins was decreased (Figure 5A). Immunoprecipitation and Western blot for FGFR3 proteins confirmed the presence of both FGFR3 proteins in the cell lysate, suggesting that this decrease in phosphorylation did not simply reflect the loss of total FGFR3IIIc and FGFR3IIIS proteins from the soluble fraction after treatment with bFGF (Figure 4A). In U266 cells that express FGFR3IIIc but not FGFR3IIIS, the FGFR3-precipitated protein was phosphorylated after exposure to bFGF (0.1–10 ng ml^−1^). Unlike in TC-32 cells, phosphorylation of FGFR3IIIc was not decreased when U266 cells were exposed to higher concentrations of bFGF (10 ng ml^−1^), suggesting the FGFR3IIIS may modulate phosphorylation of FGFR3IIIc. This requires further investigation. The treatment of U266 cells with bFGF had no effect on the subcellular localisation of FGFR3IIIc (results not shown).

The exposure of TC-32 cells to bFGF (0.1–10 ng ml^−1^) or aFGF (0.1–10 ng ml^−1^) induced phosphorylation of FGFR1-immunoprecipitated proteins (Figure 5B), and phosphorylation appeared greatest after exposure to bFGF rather than aFGF. The treatment of TC-32 cells with either bFGF or aFGF resulted in activation of the intracellular signalling protein ERK (Figure 5C).

### Knockout of FGFR3IIIS is associated with growth arrest of TC-32 cells *in vitro*

**Figure 6:**
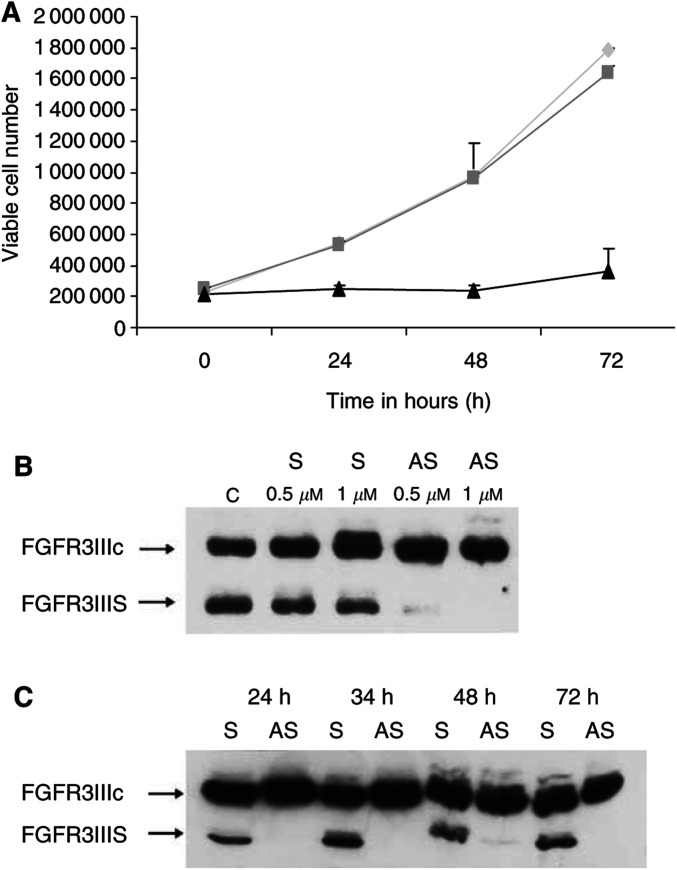
Knockout of FGFR3IIIS is associated with a decrease in viable TC-32 cell number *in vitro* (**A**) TC-32 cells treated with FGFR3IIIS antisense (▴) in the presence of reduced FCS showed reduced viable cell number 48 and 72 h after addition of antisense compared to TC-32 cells treated with a random scrambled 24-mer oligonucleotide (
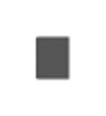
; *p*<0.001) or cells cultured under normal growth conditions, that is, no oligonucleotide (
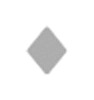
; *p*<0.001). (**B**) Antisense FGFR3IIIS (AS; 0.5 and 1 *μ*M) reduced the level of FGFR3IIIS expression 48 h after treatment compared to expression in TC-32 cells treated with a random scrambled oligonucleotide (S; 0.5 and 1.0 *μ*M) or no oligonucleotide (**C**). Antisense FGFR3IIIS (AS; 1 *μ*M) or the random scrambled oligonucleotide (S; 1 *μ*M) was added to the TC-32 cell cultures at 0, 24 and 48 h. FGFR3IIIS expression was inhibited in cultures treated with antisense FGFR3IIIS (AS) compared to those treated with the random scrambled oligonucleotide(S) at 24, 34, 48 and 72 h.

Wild-type TC-32 cells express FGFR3IIIS, detected by Western blot (Figure 4A). Random scrambled 24-mer oligonucleotides actively taken up by TC-32 cells had no effect on growth (determined by counting viable cell number) of wild-type TC-32 cells (Figure 6A

). However, delivery of an antisense FGFR3IIIS 24-mer (1 *μ*M) over three consecutive days was associated with a decrease in viable TC-32 cell number compared to that in cell cultures grown under normal growth conditions (at 48 h *P*<0.001, q=13.3; at 72 h *P*<0.001, *q*=20.2) or in the presence of the random scrambled oligonucleotide (at 48 h *P*<0.001, *q*=13.1, at 72 h *P*<0.001, *q*=18.0) (Figure 6A). The FGFR3IIIS protein was preferentially decreased compared to FGFR3IIIc 24, 48 and 72 h after incubation with the antisense FGFR3IIIS 24-mer at 1 *μ*M (Figure 6B). In the presence of the random scrambled 24-mer oligonucleotide (1 *μ*M for 48 h), FGFR3IIIS and FGFR3IIIc proteins were detected. These observations suggest that FGFR3IIIS may act as a dominant negative, inhibiting FGFR3-induced growth arrest and possibly differentiation. This hypothesis requires further investigation.

## DISCUSSION

Using RT–PCR a novel splice variant of FGFR3, missing 30 base pairs within exon 7 encoding a region between the second and third Ig-like loops of the extracellular domain (this contains a potential glycosylation site), the second half of the third Ig-like loop (including a disulphide bond site) and the transmembrane domain has been identified. The sequence encoding the protein kinase domains of this receptor is identical to that of wild-type FGFR3. An FGFR3 splice variant similar to that of FGFR3IIIS, with a deletion (bases 933–1269) overlapping that of FGFR3IIIc (bases 969–1315), has previously been described in normal breast epithelial and breast tumour cell lines (Johnston *et al*, 1995). This deletion was reported to result in loss of exons 7 and 8. Unfortunately, this splice variant was not fully sequenced so it is not possible to compare it directly with that of FGFR3IIIS or the published FGFR3 mRNA sequence (accession number M58051). Unlike recently described nonsense transcripts of FGFR3 (Jang *et al*, 2000), the coding region of FGFR3IIIS remains in-frame resulting in a novel FGFR3 protein product that is expressed in the membrane and soluble cell fractions.

The expression of this novel protein FGFR3IIIS in the soluble fraction is consistent with previous studies suggesting that deletion of the transmembrane domain of FGFR3 generates a soluble form of the receptor (Johnston *et al*, 1995). However, FGFR3IIIS is predominantly expressed in the membrane fraction under normal growth conditions, suggesting a potential role in regulating membrane-associated FGFR3 activity. Unexpectedly in TC-32 cells the full-length wild-type FGFR3IIIc, in which the transmembrane coding region remains intact, was expressed predominantly in the soluble fraction suggesting modification of its expression in the ESFT cells. Whether this is effected through FGFR3IIIS and has a role in malignant transformation of ESFT remains to be seen. During the preparation of this manuscript a soluble FGFR3 isoform has been described in human SaOS-2 osteosarcoma cells (Jang, 2002). Like FGFR3IIIS, skipping of exons 8, 9 and 10 generated an mRNA encoding an FGFR3 in which the COOH-terminal portion of the IG-like III and transmembrane domains are deleted. The significance of the additional 30 bp deletion in exon 7 of FGFR3IIIS is not clear, although loss of the potential glycosylation site in this region suggests that it may alter glycosylation of the protein. The presence of soluble transcripts of FGFR4 have also recently been described and implicated in the modulation of FGF in MCF7 breast cancer cells (Ezzat *et al*, 2001), suggesting that soluble FGFRs may be a feature of tumour cells where they disrupt the FGF/FGFR function.

The disrupted regulation of mRNA processing is a feature of neoplastic transformation (Haber *et al*, 1993; Yamaguchi *et al*, 1994; Sager, 1997), consistent with the high frequency of FGFR3IIIS transcripts detected in tumour but not normal cells. Alternatively, spliced variants in tumour cells are usually rapidly degraded through the so-called nonsense-mediated decay pathway, to prevent the synthesis of incomplete and potentially harmful proteins. However, in some genetic diseases the nonsense-mediated decay pathway is inactivated, stabilising the alternatively spliced transcripts (Culbertson, 1999). In colorectal cancer, FGFR3 transcripts are readily detected by RT–PCR (Jang *et al*, 2000); these may have escaped the nonsense-mediated decay pathway or may be the breakdown products from larger RNA transcripts, although a frame-shift means that they do not produce protein products. In contrast, the FGFR3IIIS transcript described in this paper has an intact open-reading frame encoding a protein product detected by Western blot using an FGFR3 antibody to the COOH domain.

This novel protein appears to modulate the FGFR3 gene function. The decreased growth of TC-32 cells following knockout of FGFR3IIIS is consistent with the hypothesis that FGFR3IIIS may compete with FGFR3 and/or its variants to antagonise their function, for example, growth arrest and differentiation. These observations are consistent with the putative role of FGFR3 as a regulator of differentiation (Govindarajan and Overbeek, 2001; Ornitz, 2001), and previous studies suggesting FGFR's lacking a transmembrane domain disrup the FGFR gene function *in vivo* through a dominant-negative mechanism (Peters *et al*, 1994; Celli *et al*, 1998). The decreased expression of FGFR3 variants in the soluble fraction after exposure to bFGF implies that FGFR3IIIS may regulate FGF trafficking within the cell or act as a negative feedback mechanism sequestering FGF away from cell surface receptors; on the other hand, alternative splicing in the Ig-like domain III may create receptors with different ligand-binding preferences (Chellaiah *et al*, 1994; Lin *et al*, 1997). Further studies are required to investigate these possibilities. As with other tyrosine kinase receptors, FGFRs are activated by dimerisation resulting in autophosphorylation and subsequent recruitment of intracellular signalling proteins. Preliminary evidence supports the hypothesis that FGFR3IIIS may modulate the activation and trafficking of other FGFRs, although remaining unphosphorylated itself. These hypotheses and characterisation of ligand interactions, including those of the most selective FGFR3 ligand FGF8, require further investigation.

In summary, we have described alternative splicing of FGFR3 in the third Ig-like loop of the extracellular domain to generate a novel spliced variant of FGFR3IIIc, FGFR3IIIS, frequently expressed in tumour but rarely in normal cells. This appears to code for a receptor that may act as a dominant negative to modulate the activation and trafficking of FGFs and FGFRs, influencing cell growth and phenotype. Our results support the hypotheses that alternative splicing of the FGFR3 Ig-domain III may contribute to malignant transformation, and represents a mechanism for the generation of receptor diversity.
